# Neuroregulatory Effects of Microcone Patch Stimulation on the Auricular Branch of the Vagus Nerve and the Prefrontal Cortex: A Feasibility Study

**DOI:** 10.3390/jcm13082399

**Published:** 2024-04-20

**Authors:** Akihiro Kawasaki, Yutaka Matsuzaki, Taku Kawada

**Affiliations:** 1 College of Social Csciences, Ritsumeikan University, Kyoto 603-8577, Japan; 2 Graduate School of Education, Tohoku University, Sendai 980-8576, Japan; t-kawada@sendaishirayuri.net; 3 Institute of Development, Aging and Cancer (IDAC), Tohoku University, Sendai 980-8575, Japan; yutaka.matsuzaki.e5@tohoku.ac.jp; 4 Sendai Shirayuri Gakuen Elementary School, Sendai 981-3205, Japan

**Keywords:** autonomic nervous activity, microcone patch stimulation, dorsolateral prefrontal cortex

## Abstract

**Background**: The primary purpose of this study was to preliminarily examine the effects of autonomic nervous system activity on the dorsolateral prefrontal cortex. Recent studies have examined approaches to modulating autonomic activity using invasive and non-invasive methods, but the effects of changes in autonomic activity during cognitive tasks on the dorsolateral prefrontal cortex have not been fully investigated. The purpose of this preliminary investigation was to examine changes in autonomic activity and blood oxygen saturation in the dorsolateral prefrontal cortex during reading tasks induced by vagus nerve stimulation using a microcone patch. **Methods**: A cohort of 40 typically developing adults was enrolled in this study. We carefully examined changes in autonomic nervous system activity and blood oxygen saturation in the dorsolateral prefrontal cortex during a reading task in two conditions: with and without microcone patch stimulation. **Results**: Significant changes in brain activation in the dorsolateral prefrontal cortext due to microcone patch stimulation were confirmed. In addition, hierarchical multiple regression analysis revealed specific changes in reading task-related blood oxygen saturation in the dorsolateral prefrontal region during microcone patch stimulation. **Conclusions**: It should be recognized that this study is a preliminary investigation and does not have immediate clinical applications. However, our results suggest that changes in autonomic nervous system activity induced by external vagal stimulation may affect activity in specific reading-related regions of the dorsolateral prefrontal cortex. Further research and evaluation are needed to fully understand the implications and potential applications of these findings.

## 1. Introduction

For depression and anxiety, pharmacotherapies, such as selective serotonin reuptake inhibitors, and psychotherapies, such as cognitive behavioral therapy, are gaining evidence regarding their efficacy [1,2,3]. Simultaneously, however, they are costly and inaccessible to many individuals [4,5,6]. Therefore, noninvasive, low-cost, and accessible alternatives are also being investigated. For example, relaxation techniques, such as progressive muscle relaxation, have been studied [7]. These techniques are often used as part of psychological interventions in conjunction with psychotherapy to act on the autonomic nervous system [8], which is closely related to anxiety.

More recently, approaches that use external devices and stimuli to control autonomic nervous system activity have emerged. Such approaches include transcutaneous auricular vagus nerve stimulation (taVNS) [9,10] and dermal stimulation with microcone patches [11,12]. taVNS involves placing electrodes on the auricle to deliver weak electrical stimulation to the auricular vagus nerve, which is directly connected to the vagus nerve. taVNS stimulates the parasympathetic nervous system and consequently inhibits the sympathetic nervous system via the auriculovagal branch of the vagus nerve [13].

Although taVNS has been reported to inhibit sympathetic activity and the brainstem effects of stimulation-induced fluctuations on autonomic nervous system activity [14,15], the findings about its effects on the central nervous system and its efficacy in various conditions are mixed, due to factors such as patients’ characteristics, the equipment used, and its setting [10,16,17]. Finally, taVNS has been shown to have effects on depression as demonstrated by meta-analyses [18]. However, taVNS requires specialized techniques and equipment [10], and its mechanism of action is still in the process of elucidation.

In contrast, microcone patch skin stimulation directly stimulates the skin using a patch densely packed with extremely fine projections [11,12,19] and is suggested to change autonomic response or brain activity. Unlike taVNS, microcone patches do not require specialized techniques or equipment, and the patch itself is not considered a medical procedure, making it suitable for self-medication [12]. In a study on chronic pain, the microcone patch was reported to suppress the “slow” reflex involved in the emotional aspect of pain in the somatosensory nerves by up to 60% [19,20]. The suppression of the cardiac sympathetic reflex by microcone patches at rest has been found to affect the anterior cingulate gyrus [21].

In a preliminary study, we investigated variations in autonomic activity following the application of a microcone patch to the auricle, confirming that autonomic responses vary with stimulation [22]. If stimulation can regulate autonomic activity, it is anticipated that the activity of the limbic system, which is known for its inhibitory influence on autonomic activity [23,24], would also adjust in response. Furthermore, given the established antagonistic relationship between the medial and lateral prefrontal cortices [25], the effects are posited to eventually resonate within the lateral prefrontal cortex. 

As mentioned above, it has been demonstrated that microcone stimulation alters the autonomic nervous system response and neural activity. However, previous studies have focused on responses during rest, and the effects of auricular stimulation with microcone patches during tasks remain unclear. Therefore, this study examined whether the autonomic nervous system influences brain activity during tasks through the use of microcone patches. If effective control of autonomic activity during tasks becomes achievable by stimulating the auricular branch of the vagus nerve using a microcone patch, this would be potentially beneficial for subjects coping with task-related stress.

Based on preliminary findings [22], we hypothesize that the regulation of autonomic activity through stimulation of the auricular branch of the vagus nerve might extend its influence to prefrontal cortex activity during tasks. To verify this, it was essential not to adopt simple tasks but to prepare tasks that involve multiple problem-solving strategies. In this study, we adopted a short-text reading comprehension task. It has been suggested that there is a link between reading, the strategies for that purpose, and the autonomic activity during this process [26,27]. In a reading comprehension task, when employing a bottom-up reading strategy, areas, including the frontal eye field, in the dorsolateral prefrontal cortex are activated [28,29]. Moreover, when decoding is slightly difficult to perform, using visuospatial working memory is common, which significantly activates the right dorsolateral prefrontal cortex [30]. In other words, typical reading activities do not require considerable activation of the right dorsolateral prefrontal cortex, and compensatory activation in this area is thought to be eliminated by the redistribution of cognitive resources [31].

## 2. Materials and Methods

### 2.1. Participants

Forty-three healthy students belonging to institutions of higher education were recruited through the institutes’ part-time job application system. The participants confirmed that they were right-handed and had no history of cerebrovascular, psychiatric, or ocular diseases through their self-reports as the eligibility criteria. They read a description of the study and provided informed consent. Three participants with missing data were excluded from the analysis; therefore, 40 healthy adults (25 males and 15 females) were finally included in this study. The mean overall age was 22.43 ± 3.22 years; 22.23 ± 1.50 years in males; and 22.79 ± 5.15 years in females. The experiment was performed according to the Declaration of Helsinki, and the study protocol was approved by the Research Ethics Committee of Tohoku University Kawauchi South District Medical Research on Human Subjects (No. 2020-6, approval date: 24 September 2020). 

### 2.2. Task and Procedure

Two tasks—a short-text comprehension task and a reading comprehension task—were employed to evoke mental activity associated with prefrontal cortex function. The task was presented on a 28-inch monitor using PowerPoint in a room for psychological experiments at universities. The participants were instructed to adjust their chairs to modify the distance from the monitor and determine the most comfortable position. Subsequently, they were equipped with a pulse wave meter and near-infrared spectroscope (NIRS).

**Figure 1:**
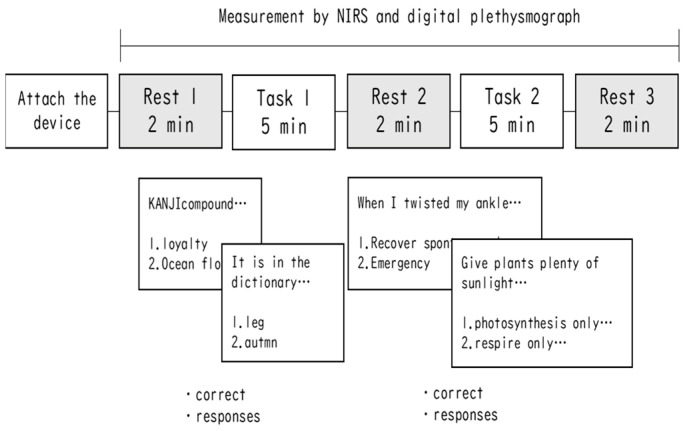
Experimental procedure.

A schematic summary of this experimental procedure is shown in Figure 1. The experiment was performed with and without auricular stimulation with microcone patches, with one set of each condition separated by 1 week (within-subject block design). The order of each condition was randomized for each participant. Each set comprised a 2 min rest condition, followed by a 5 min task condition (block 1), then a 2 min rest condition, followed by a 5 min task condition (block 2), and finally a 2 min rest condition.

In the rest condition, the participants were instructed to relax and not think about anything while keeping their eyes open. In the task condition, the participants were instructed to answer general knowledge questions. They were required to answer the questions by operating the mouse using their dominant hand, which was not holding the pulse wave meter probe. All questions were five choice, with the difference between tasks 1 and 2 being that the choices were words (e.g. In the dictionary, it is appropriate to select one as the earliest one to appear. 1. Leg, 2. Autumn, 3. Bubble, etc.) or sentences (“When I twisted my ankle”, select an inappropriate response. 1. “Recover spontaneously, so it’s fine to leave it alone without particular concern”…, etc.). The questions for each were developed with reference to the Japanese language test on sentence comprehension [31,32]. Each task consisted of 30 questions presented randomly over a 5 min period. After answering one question, the next question was presented, and the subjects were taught to answer as quickly as possible within the time available. The difficulty of each block was adjusted to be uniform.

The average duration of the experiment, including obtaining informed consent and setting up the measurement of physiological indices for each participant, was approximately 30 min. All participants, including those who were excluded, were compensated after the experiment.

### 2.3. Characteristics of the Microcone Patch

**Figure 2:**
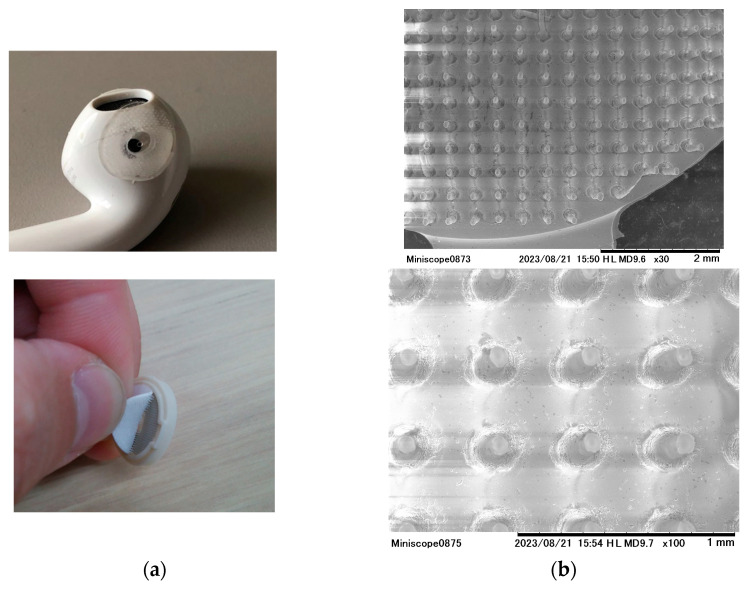
Microcone patch. (**a**) A microcone patch is applied to earbuds to stimulate the posterior wall of the ear canal. (**b**) Enlarged view of the microcone patch. The flattened tip prevents puncture.

For the skin stimulation method, we used a microcone patch. This patch comprises an 11 mm diameter circular base lined with 500 nanometer high silicon projections (Figure 2a). A microcone patch was fabricated with a diameter of 11 mm and a circular base, featuring 650 evenly distributed silicone protrusions, each with a height of 800 microns, a width of 50 microns, and a flat tip (Figure 1). Due to the need for a specialized mold for its creation, the production of the microcone patch leveraged a product by TOYORESIN Corporation, Ltd., known for its experience in manufacturing such patches. A notable feature of the microcone patch is its flexible material and the presence of minute protrusions. Moreover, the flat-tipped design ensures that while it stimulates the skin, it does not pierce into it. A previous study comparing microcone patches suggests that it is important that the silicone protrusions vibrate while in contact with the skin [19]. Skin stimulation via the microcone patch was achieved by initially attaching the patch to the inner earphone (Figure 2b) and then snugly securing the patched earphone. For the non-stimulation condition, a disk-shaped patch of the same size and material, but without the protrusions, was used, serving as a control.

### 2.4. Measurement of Autonomic Nervous System Activity

TAS-9view (YKC.Ink, https://ykcgroup.com/ (accessed on 4 March 2024)) was used to monitor the pulse wave of the participants. Note that the probe of the pulse wave meter was attached to the index finger of the non-dominant hand. Each autonomic nervous system index was calculated, monitored, and recorded within the device from the acquired pulse waves during the rest periods, as well as the task conditions. Specifically, pnn50 (%) was used to reflect vagal nerve tension and LF/HF was used to reflect sympathetic nerve tension, with both determined by pulse wave. The vagal tone index, pnn50 (%), was calculated by counting NN50 (intervals between heartbeat peaks exceeding 50 msec).

### 2.5. NIRS Recording and Preprocessing

**Figure 3:**
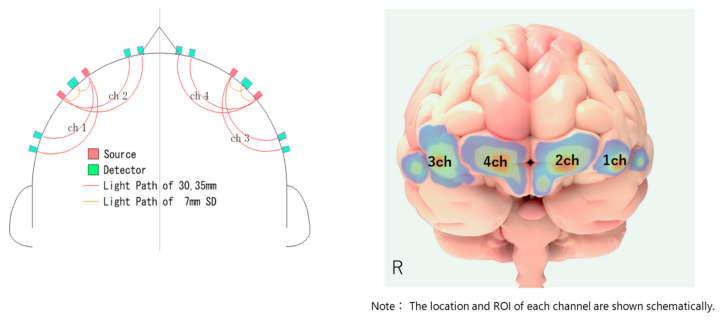
NIRS setup.

The NIRS used in this study was a 4 CH wireless device produced by Astem (Hb131S; https://astem-jp.com/en/ (accessed on 4 March 2024)). NIRS setup is visualized in Figure 3. First, the subject’s Fpz was identified based on the international 10–20 system. Then, the Fpz was positioned between CH_2_ and CH_4_. The NIRS sampling rate was set at 10 Hz, and the values of oxygenated hemoglobin (OxHb) observed during each task and resting period were used as indices. Skin blood flow was also monitored during the tasks to account for its effects. To reduce noise as much as possible, a moving average was set to ‘3’ in the software.

### 2.6. Statistical Analysis

The signals representing the degree of OxHb from NIRS were corrected by subtracting the mean signal value during rest, and then, the averaged values during two tasks were computed for each subject/condition. Initially, the descriptive statistics of each indicator between conditions (with stimulation vs. non-stimulation) were compared using paired t-tests. To investigate the differential effects of the microcone patch with and without stimulation, a hierarchical multiple regression analysis was performed. Mean OxHb concentration from NIRS channels 1–4, which represent the activity of the lateral prefrontal cortex during the task, served as the dependent variable. Conversely, pnn50 (%) and LF/HF, indices of autonomic activity, were used as independent variables. Statistical analyses were performed using Python (ver. 3.9.12) and HAD (ver. 15.00). A threshold of *p* < 0.05 was set for determining statistical significance.

## 3. Result

### 3.1. Descriptive Statistics and Differences by Condition

The descriptive statistics are summarized in Table 1. The mean concentration of OxHb for CH1 was significantly higher in the stimulated condition (M ± SD = 0.022 ± 0.068) compared with the unstimulated condition (M ± SD = −0.006 ± 0.041, *t*(64.377) = 2.25, *p* = 0.028). In contrast, the mean concentration of OxHb for CH4 was significantly higher in the unstimulated condition (M ± SD = 0.009 ± 0.041) than in the stimulated condition (M ± SD = −0.018 ± 0.074). There were no statistically significant differences in the mean concentration of OxHb for CH_2_, CH_3_, LF/HF ratio, and pnn50 (%). The accuracy tended to be higher in the stimulated condition (M ± SD = 0.920 ± 0.056) than in the unstimulated condition (M ± SD = 0.895 ± 0.073), but it was not statistically significant (*t*(73.293) = 1.673, *p* = 0.099).

### 3.2. Hierarchical Regression Analysis

#### 3.2.1. Patch-Stimulation Group

The results of the hierarchical multiple regression analysis under the stimulated condition are summarized in Table 2. In CH_2_ and CH_3_, the coefficient of determination of step 3 is statistically significant (CH2: *F*(3, 36) = 3.299, *p* = 0.031, *R*^2^ = 0.216; CH3: *F*(3, 36) = 4.214, *p* = 0.012, *R*^2^ = 0.198), while channel 1 showed a significant tendency (*F*(3, 36) = 2.542, *p* = 0.072, *R*^2^ = 0.175). In CH1, the effect of the LF/HF ratio was statistically significant for the mean OxHb concentration in step 3 (Β = 0.613, *p* = 0.019). In CH_2_, the effect of LF/HF ratio-by-pnn50 interaction was statistically significant for the mean OxHb concentration in step 3 (Β = 0.520, *p* = 0.037). In CH_3_, the effect of pnn50 (%) (Β = 0.519, *p* = 0.028) and LF/HF ratio-by-pnn50 interaction (Β = 0.756, *p* = 0.003) was statistically significant for the mean OxHb concentration in step 3. The coefficient of determination for CH_4_ was not statistically significant (*F*(3, 36) = 1.088, *p* = 0.367, *R*^2^ = 0.083) in step 3, and none of the explanatory variables were statistically significant either.

#### 3.2.2. Non-Stimulation Group

There were no statistically significant models or independent variables noted across CH1 to CH4 in step 3 (Table 3).

## 4. Discussion

The aim of the present study was to determine whether changes in autonomic nervous system activity induced by the application of a microcone patch on the auricular branch of the vagus nerve affect activity in the lateral prefrontal cortex. The results showed differences in blood oxygen saturation in some CHs with and without stimulation and further showed that autonomic indicators influenced blood oxygen saturation indices in the lateral prefrontal cortex under the condition of a sentence comprehension task only, stimulated by the microcone patch. Previous studies have shown that ‘soft’ skin stimulation with microcone patches suppresses cardiac sympathetic activity and that this change affects resting activity in the anterior cingulate gyrus [21]. The present study is the first to demonstrate changes in the relationship between autonomic nervous system activity and pre-frontal hemodynamics during work with stimulation by microcone patches.

### 4.1. Effects of Simulation on the Cerebral Cortex via Changes in Autonomic Dictation Paths

Hierarchical multiple regression analysis with blood oxygen saturation in each channel of NIRS in the lateral prefrontal cortex as the dependent variable, LF/HF as a measure of sympathetic tone, and pnn50 (%) as a measure of vagal tone and interaction as independent variables showed that without stimulation with the microcone patch, significant results could not be obtained in all channels. However, during microcone stimulation, the results were significant in the posterior left dorsolateral prefrontal cortex (1CH and 2CH) and posterior right dorsolateral prefrontal cortex (3CH). In CH1, the effect of LF/HF was significant; in CH_2_, the interaction term between LF/HF and pnn50 (%) was significant; and in CH_3_, both the effect of pnn50 (%) and the interaction term between LF/HF and pnn50 (%) were significant. However, there was no statistically significant effect on 4CH.

Although identifying a specific anatomical region in CH_1_ is difficult because of the small number of channels, it is thought to reflect activity in the posterior left dorsolateral prefrontal cortex, which is a large region of language processing. This region is associated with language processing in general and cognitive control in particular and is centrally activated during reading tasks [29]. Conversely, CH_4_ showed increased brain activity during sentence comprehension in the non-stimulus condition compared to the stimulus condition. This region (right anterior DLPFC) is involved in visuospatial working memory. It has been suggested that compensatory reading strategies secondarily activate this region, particularly when the subject has difficulty in reading comprehension [30,33]. In our results, it is suggested that the stimulation in the patch changed brain activity so that compensatory brain activity was reduced and the original brain activity used in the reading task was increased. The remaining channels (CH_2_ and CH_3_) may have been areas less sensitive to reading and compensatory working memory use, but it cannot be ruled out that they may have just been underpowered due to the insufficient sample size in our study.

The hypothesis of this study is supported by the finding that the autonomic index of activity could affect the index of blood oxygen saturation in the lateral prefrontal cortex at specific CHs only during microcone patch stimulation. Regarding the effects on the old cortex reported in the previous study, a positron emission tomography study of microcone patches and a placebo applied to both cheeks reported significantly higher metabolism during stimulation in the anterior cingulate cortex; however, no significant differences were found in the somatosensory or insular cortices [21]. However, these experiments were performed under resting conditions and stimulation within physically and ethically acceptable limits and did not examine the effects of purposeful mental activity or specific anxiety or stress. Fluctuations in autonomic activity in everyday situations are commonly induced by exposure to anxiety and various stresses (e.g., workload and stress load), and if stimulation with the microcone patch acts on task- or situation-induced autonomic activity, it may affect the medial or lateral prefrontal cortex. Therefore, it is reasonable to assume that if microcone patch stimulation acts on task- or situation-induced autonomic activity, it will also affect the medial and lateral prefrontal cortices. Recently, the increasing number of reports on the effects of autonomic activity suppression on the central nervous system through various methods can also be seen as further supporting the directionality of this research [34]. Future clinical applications should consider various actual work situations and subjects.

### 4.2. Background and Mechanism of Action

Studies have suggested that microcone patch stimulation operates by inducing the production of endogenous opioids [21,35]. These naturally occurring opioid peptides are synthesized within the body as a result of physiological processes. Notably, microcone patch stimulation appears to inhibit the cardiac sympathetic reflex in a manner similar to the effects of morphine. This inhibitory response is nullified when opioid receptor blockers are introduced, which strongly underscores the pivotal role of the opioid system in this mechanism. The potency of the reflex-suppressing effect of microcone patches is significant, with some reports indicating its efficacy to be approximately 60% of that exhibited by morphine, an external source of opioids [19].

Additionally, recent research [13] has suggested that taVNS, such as that delivered through microcone patches, also influences the activation of the insular cortex. The insular cortex is notably rich in opioid receptors. Moreover, it serves as a central hub within the salience network, which orchestrates various cognitive functions. Previous studies have shown improved cognitive test performance requiring salience network and frontal lobe function as a result of using taVNS [35,36]. Auricular stimulation with the microcone patch may also possibly affect this performance, although the mechanism has not been found to be exactly the same as with taVNS.

### 4.3. Potential Clinical Applications

Our study has illuminated the influence of auricular stimulation using microcone patches on autonomic responses and frontal lobe activity during work-related tasks. Nevertheless, as we contemplate its broader clinical applications, there are essential considerations and challenges to address.

Primarily, understanding the microcone patch as a coping mechanism, not a foundational treatment, is crucial. Detailed evaluations of its efficacy, the longevity of its effects, and the potential for users to become acclimated to the stimulation are required. Moreover, evidence suggests that the effectiveness of the microcone patch varies based on both the site and intensity of its application [37]. Ensuring consistent and reproducible results is a pivotal concern.

However, the potential applications of microcone patches remain substantial. Their non-invasive nature [12] is a significant advantage, differentiating them from medical interventions, such as taVNS [10], and allowing for self-application without expert oversight. Their discreet design ensures that users can wear them without drawing undue attention. External medical devices often cause patients to suffer from associated prejudice [38]. However, the microcone patch used in this study may be easier for many to use because of its less conspicuous design.

Given these attributes, microcone patches might serve as invaluable self-administered tools for stress and anxiety relief. They could offer solace to individuals with mild anxiety or mood disturbances that do not warrant a clinical diagnosis. Additionally, their use in specific situations, such as aiding stutterers in conversations or dyslexics during reading, underscores their potential therapeutic value; this is because the stress involved in speaking and reading in these disorders amplifies their difficulties [39,40].

### 4.4. Limitations

Although our study indicates the potential effects of microcone patch stimulation on the activation of the dorsolateral prefrontal cortex during reading, several limitations persist. First, this study predominantly relied on NIRS-measured indices related to the dorsolateral prefrontal cortex and autonomic nervous system. To truly discern the mechanistic influence of the microcone patch, a more holistic brain imaging approach, particularly one that encompasses regions, such as the limbic system and medial prefrontal cortex, is warranted. Although NIRS was used as the method of estimating brain activity in this study, it may be useful to use more improved methodology such as head circumference reporting and registration using anatomical images from MRI to improve the accuracy of estimating the anatomical location of the region of interest. Second, autonomic nerve activity fluctuations can vary significantly based on work situations and individual perceptions. The effect may be attenuated in scenarios with lower task demands or when subjects perceive tasks as less challenging. Second, fluctuations in autonomic activity can be highly dependent on the task situation and individual perceptions. Effectiveness may be attenuated in scenarios where the task is less demanding or when subjects perceive the task to be less difficult. Although the pulse wave system was used in this study, it may be important to follow up with a combination of other methods, such as electrocardiogram. Third, the participants were healthy college students. To ensure the generalizability of our findings, it is essential to replicate the data in diverse demographic populations, including preclinical and patient populations, as well as in populations with different ages, genders, and various cognitive/psychological characteristics. Fourth, it is vital to recognize that the efficacy of the microcone patch is rooted in its array of microprojections. Broad stimulation of the same area without these microprojections will not replicate the observed effects [19]. It would be useful to accumulate studies on the characteristics of microcone patches themselves using various variations.

## 5. Conclusions

The present study identified effects on the dorsolateral prefrontal cortex of fluctuations in autonomic nervous system activity induced by skin stimulation with microcone patches. The effects indicate the potential for appropriate redistribution of cognitive resources associated with this activity. The microcone patch is a versatile tool for influencing autonomic activity and is expected to be applied to stress reduction and other applications in the future.

## Figures and Tables

**Table 1:** Descriptive statistics.

Index	With Stimulation (M ± 1SD)	Without Stimulation (M ± 1SD)	Statistics
CH1	0.022 ± 0.068	−0.006 ± 0.041	*t*(64.377) = 2.25, *p* = 0.028
CH2	−0.017 ± 0.104	0.013 ± 0.061	*t*(63.06) = −1.549, *p* = 0.127
CH3	0.015 ± 0.090	−0.006 ± 0.047	*t*(59.146) = 1.329, *p* = 0.189
CH4	−0.018 ± 0.074	0.009 ± 0.041	*t*(60.99) = −2.017, *p* = 0.048
LF/HF ratio	1.450 ± 1.146	1.530 ± 0.843	*t*(71.665) = −0.355, *p* = 0.724
pnn50 (%)	16.486 ± 15.774	18.858 ± 17.565	*t*(77.115) = −0.635, *p* = 0.527
Accuracy	0.920 ± 0.056	0.895 ± 0.073	*t*(73.293) = 1.673, *p* = 0.099

**Table 2:** Hierarchical multiple regression analysis results for “with stimulation”.

CH	Step	Independent Variables	B	95% CI for B		Se B	β	*p* of β	R^2^	ΔR^2^
1CH	Step 1								0.133 *	
		Intercept	0.022	0.002	0.043	0.010				
		LF/HF	0.022	0.004	0.040	0.009	0.365	0.021		
	Step 2								0.145 ^†^	0.012
		Intercept	0.022	0.002	0.043	0.010				
		LF/HF	0.024	0.005	0.044	0.010	0.412	0.017		
		pnn50 (%)	0.001	−0.001	0.002	0.001	0.120	0.470		
	Step 3								0.175 ^†^	0.029
		Intercept	0.030	0.005	0.054	0.012				
		LF/HF	0.036	0.007	0.066	0.014	0.613	0.016		
		pnn50(%)	0.001	−0.001	0.003	0.001	0.319	0.193		
		LF/HF × pnn50 (%)	0.001	−0.001	0.003	0.001	0.280	0.265		
2CH	Step 1								0.112 *	
		Intercept	−0.017	−0.049	0.015	0.016				
		LF/HF	−0.030	−0.059	−0.002	0.014	−0.334	0.035		
	Step 2								0.114	0.002
		Intercept	−0.017	−0.049	0.015	0.016				
		LF/HF	−0.032	0.015	−0.001	0.015	−0.354	0.042		
		pnn50 (%)	0.000	0.001	0.002	0.001	−0.049	0.770		
	Step 3								0.216 *	0.102 *
		Intercept	0.004	−0.033	0.040	0.018				
		LF/HF	0.002	−0.042	0.046	0.022	0.022	0.928		
		pnn50 (%)	0.002	−0.001	0.005	0.002	0.319	0.181		
		LF/HF × pnn50 (%)	0.003	0.000	0.006	0.001	0.520	0.037		
3CH	Step 1								0.045	
		Intercept	0.015	−0.014	0.043	0.014				
		LF/HF	−0.017	−0.042	0.009	0.012	−0.212	0.189		
	Step 2								0.045	0.000
		Intercept	0.015	−0.014	0.044	0.014				
		LF/HF	−0.017	−0.045	0.011	0.014	−0.218	0.218		
		pnn50 (%)	0.000	−0.002	0.002	0.001	−0.016	0.927		
	Step 3								0.260 *	0.215 **
		Intercept	0.041	0.010	0.071	0.015				
		LF/HF	0.026	−0.011	0.062	0.018	0.327	0.163		
		pnn50 (%)	0.003	0.000	0.006	0.001	0.519	0.028		
		LF/HF × pnn50 (%)	0.004	0.001	0.006	0.001	0.756	0.003		
4CH	Step 1								0.046	
		Intercept	−0.018	−0.041	0.006	0.012				
		LF/HF	−0.014	−0.035	0.007	0.010	−0.214	0.185		
	Step 2								0.054	0.009
		Intercept	−0.018	−0.041	0.006	0.012				
		LF/HF	−0.016	−0.039	0.006	0.011	−0.253	0.153		
		pnn50 (%)	0.000	−0.002	0.001	0.001	−0.100	0.567		
	Step 3								0.083	0.029
		Intercept	−0.010	−0.038	0.018	0.014				
		LF/HF	−0.003	−0.037	0.030	0.017	−0.054	0.835		
		pnn50 (%)	0.000	−0.002	0.003	0.001	0.095	0.708		
		LF/HF × pnn50 (%)	0.001	−0.001	0.003	0.001	0.277	0.295		

Note. CI: confidence interval, ** *p* < 0.01 * *p* < 0.05, ^†^ *p* < 0.10.

**Table 3:** Hierarchical multiple regression analysis results for “non-stimulation”.

CH	Step	Independent Variables	B	95% CI for β		Se B	β	*p*	R^2^	ΔR^2^
1CH	Step 1								0.003	
		Intercept	−0.006	−0.019	0.007	0.007				
		LF/HF	0.002	−0.014	0.019	0.008	0.051	0.757		
	Step 2								0.005	0.003
		Intercept	−0.006	−0.020	0.008	0.007				
		LF/HF	0.001	−0.017	0.019	0.009	0.027	0.884		
		pnn50(%)	0.000	−0.001	0.001	0.000	−0.056	0.762		
	Step 3								0.066	0.061
		Intercept	−0.001	−0.016	0.014	0.007				
		LF/HF	0.005	−0.013	0.024	0.009	0.103	0.582		
		pnn50(%)	0.000	−0.001	0.001	0.000	0.041	0.830		
		LF/HF × pnn50(%)	0.001	0.000	0.002	0.001	0.264	0.134		
2CH	Step 1								0.009	
		Intercept	0.013	−0.007	0.033	0.010				
		LF/HF	−0.007	−0.031	0.017	0.012	−0.094	0.562		
	Step 2								0.010	0.001
		Intercept	0.013	−0.007	0.033	0.010				
		LF/HF	−0.008	0.013	0.019	−0.008	−0.110	0.548		
		pnn50(%)	0.000	0.001	0.001	0.000	−0.036	0.845		
	Step 3								0.018	0.008
		Intercept	0.010	−0.012	0.033	0.011				
		LF/HF	−0.010	−0.038	0.018	0.014	−0.137	0.477		
		pnn50(%)	0.000	−0.002	0.001	0.001	−0.070	0.722		
		LF/HF × pnn50(%)	0.000	−0.002	0.001	0.001	−0.093	0.602		
3CH	Step 1								0.000	
		Intercept	−0.006	−0.022	0.009	0.008				
		LF/HF	0.000	−0.018	0.018	0.009	0.000	1.000		
	Step 2								0.000	0.000
		Intercept	−0.006	−0.022	0.009	0.008				
		LF/HF	0.000	−0.021	0.021	0.010	−0.001	0.997		
		pnn50(%)	0.000	−0.001	0.001	0.000	−0.002	0.993		
	Step 3								0.029	0.029
		Intercept	−0.002	−0.020	0.015	0.009				
		LF/HF	0.003	−0.019	0.025	0.011	0.052	0.784		
		pnn50(%)	0.000	−0.001	0.001	0.001	0.065	0.737		
		LF/HF × pnn50(%)	0.001	−0.001	0.002	0.001	0.183	0.305		
4CH	Step 1								0.000	
		Intercept	0.010	−0.004	0.023	0.007				
		LF/HF	0.000	−0.016	0.016	0.008	0.009	0.956		
	Step 2								0.000	0.000
		Intercept	0.010	−0.004	0.023	0.007				
		LF/HF	0.000	0.009	0.018	0.009	0.004	0.982		
		pnn50(%)	0.000	0.000	0.001	0.000	−0.011	0.953		
	Step 3								0.022	0.022
		Intercept	0.013	−0.003	0.028	0.007				
		LF/HF	0.002	−0.016	0.021	0.009	0.050	0.792		
		pnn50(%)	0.000	−0.001	0.001	0.000	0.048	0.807		
		LF/HF × pnn50(%)	0.000	−0.001	0.002	0.001	0.160	0.371		

Note. CI: confidence interval.

## Data Availability

The raw data supporting the conclusions of this article will be made available by the authors, without undue reservation.
